# *In vivo* Photometry during Movement Changes Our Understanding of the Direct and Indirect Pathways

**DOI:** 10.3389/fneur.2013.00118

**Published:** 2013-08-14

**Authors:** John McKinley, Allan McCarthy, Timothy Lynch

**Affiliations:** ^1^Department of Neurology, Dublin Neurological Institute at the Mater Misericordiae University HospitalDublin, Ireland

The role of the direct and indirect pathways in movement and their dysfunction in the pathophysiology of most movement disorders is difficult to grasp for clinicians. The current model whereby the direct pathway has a role akin to the accelerator when driving and the indirect to the brake makes sense at a basic level for many disorders of movement. However, some clinical phenomenology such as hemiballismus are difficult to reconcile with the current model.

Cui et al. (1), using an elegant experimental paradigm employing photometric methods *in vivo* in a mouse model have observed the moving brain at work and have attempted to disentangle the complex network of spiny projection neurons involved in the direct and indirect pathways.

The authors reflect on the prevailing model whereby two distinct projection circuits composed of spiny projection neurons in the striatum act in an opposing fashion to either promote movement (direct pathway) or inhibit movement (indirect pathway). The anatomical location of these pathways is the striatum. Surmeier in an accompanying editorial further identifies the striatum as the seat of “past experiences” and when “asked” by cortex what way to proceed, the current model which he identifies as intrinsically binary sends “go” or “no-go” instructions to cortex, via the thalamus (2).

Cui et al. reflect on the fact that despite increased understanding of basal ganglia circuitry, we still do not understand the physiological relationship and interactions between these pathways. To do so we need to observe the activity of the direct and indirect pathway spiny projection neurons in “behaving” animal models (1). However, it is difficult to distinguish direct and indirect spiny projection neuron firing using electrophysiological recordings and antidromic stimulation is unreliable and difficult due to “cross talk” between axons of both pathways (1).

The authors highlight that optogenetic technology *in vivo* facilitates the identification of specific cell types and the monitoring of activity of specific cell types (1). Limitations of this technology include the requirement for head restraint, which limits the study of structures involved in movement and the technology does not penetrate to subcortical structures therein limiting the ability to study the basal ganglia (1).

Cui et al. developed an experimental paradigm utilizing *in vivo* photometry to optically record neural activity within the striatum during free movement in a mouse model (1). They implanted a fiber-optic probe to deliver laser pulses and record emitted photons in the dorsal striatum over time. The optical indicator was a genetically encoded calcium indicator (GECI), selectively expressed in the direct pathway spiny projection neurons by injection with a recombinant viral vector to produce D1-Cre mice (2). A similar technique was used to selectively express the indicator in the indirect pathway spiny projection neurons of A2A-Cre mice (1).

Focusing specifically on their examination of the relationship between activity in the direct and indirect pathway spiny projection neurons during movement, the authors measured fluorescence whilst mice performed a lever-pressing task. They demonstrated time-locked increases in fluorescence during the initiation of the task with a similar temporal profile in both the direct and indirect pathways in all of the mice (1). They identified transient increases in fluorescence throughout the completion of different tasks and in addition during the monitoring sessions, mice were exposed to a series of active states and periods of inactivity (1).

Using the classical direct and indirect pathway model it would be logical to hypothesize that during the active periods there should be relatively more transients identified in the direct pathway spiny projection neurons compared to the indirect and during periods of inactivity there should be more transients identified in the indirect spiny projection neurons compared to the direct (1). Cui et al. identified increased activity in both direct and indirect pathways during movement and silence of both pathways during periods of inactivity. These results question the accelerator-brake metaphor applied to our current understanding of the interaction between the direct and indirect pathways. Further analysis of self-paced movement initiation toward the contralateral side demonstrated co-activation of both the direct and indirect pathways and furthermore activity in both pathways preceded movement initiation. Activity in both pathways predicted the occurrence of specific movements within 500 ms of transient initiation.

In the accompanying editorial it is suggested that rather than giving sequential “go” or “no-go” recommendations, the striatum is simultaneously making recommendations about “what to do and what not to do” (2).

Through the interrogation of the direct and indirect pathways *in vivo* and during movement, this study builds upon and changes our current understanding of the function of the basal ganglia. Whilst the results challenge the current concepts used to explain movement, further work is required including lesional studies to translate this theory into the clinical environment in order to explain the clinical phenomenology we see at the bedside.

## Authorship

Dr McKinley wrote the manuscript and Dr McCarthy and Professor Lynch reviewed and critiqued the final manuscript.
